# Effect of triiodothyronine on hyperandrogenism in women

**DOI:** 10.1186/1756-6614-6-S2-A27

**Published:** 2013-04-05

**Authors:** Gintautas Kazanavicius, I Purtokaite-Labutiniene, D Kozloviene, V Kruminis, E Kreivaitiene

**Affiliations:** 1 Lithuanian University of Health Sciences, Kaunas, Lithuania

## Introduction

The imbalance of thyroid hormones can cause the dysfunction of the reproductive system. The aim of our study is to evaluate the thyroid hormone triiodthyronine’s (T3) influence on women’s hyperandrogenism.

## Methods

42 women aged 18-35 were randomly selected with FAI > 4.5 or one of the following signs of hyperandrogenism: hirsutism, acne, menstrual disorder, with normal thyroid function. Patients were divided into two groups. The first group (n=23) received 12.5µg of T3 for 6 months and the second group (n=19) – placebo. After the 6-month course the treatment was changed accordingly. We estimated hirsutism by using the Ferriman–Gallwey score system, acne, testosterone (T) levels, sex hormone binding globulin (SHBG) and free androgen index (FAI) and we also examined ovarian volume changes.

## Results

The hirsutism score decreased from 11.86±6.17 to 8.44±5.02 (p=0.009). A significant decline of acne score was observed from 1.66 to 0.58 (p=0.02). The testosterone serum level dropped from 2.85±1.29 ng/l to 2.28±0.87 ng/l (p=0.03) and SHBG serum concentration during the 6-month period increased from 34.99±15.34 nmol/l up to 44.52±19.69 nmol/l (p≤0.03). FAI decreased from 10.11±7.29 to 6.48±4.62 (p≤0.007). The TSH level after 3 months of treatment decreased from 1.69±0.93 mIU/l to 0.82±0.69 mIU/l (p<0.001) and after 6 months was 1.15±0.9 mIU/l (p<0.01). The FT4 level dropped from 15.52 pmol/l to 10.51±2.12 pmol/l (p<0.05). The FT3 level increased from 4.73±0.69 pmol/l to 7.57±1.86 pmol/l (p<0.05). The thyroid gland volume during the treatment remained stable. The ovarian volume over 6 months changed from 25.3±9.3 to 18.36±6.25 (p=0.002). A positive correlation was found between the ovarian volume and T serum level r=0.47 (p<0.001) together with FAI level r=0.429 (p<0.001). The weight loss was observed in some of the women during the T3 treatment course. In this group the weight and ovarian volume remained stable after six months of not receiving drug treatment. Also in this group the decrease of oestrogen was seen during and over half a year after treatment. The oestrogen level for the group of women whose weight was stable or increased during the treatment, at the beginning, was lower, and during the course increased from 165.1±80 pg/ml to 205.7±99.4 pg/ml. Finally the oestrogen level significantly increased to 288.9±223.5 pg/ml (p<0.03) after six months of no T3 treatment. The difference in ovarian volume between these two groups according to the weight changes was statistically proven p=0.008. The ovarian volume decreased only during the treatment of women with the same or bigger weight, compared with the group with weight loss where the ovarian volume remained smaller after discontinuation of the treatment. Also the T3 impact on androgens after discontinuation of treatment was determined only for women with weight loss.

## Conclusions

Hirsutism, acne, androgens and ovarian volumes are decreased due to the increase of the triiodothyronine level in blood of women with hyperandrogenism signs. The long-term (6 months) clinical effect of T3 is observed in women with weight loss during treatment.

